# Changes in the skin characteristics of Nellore steers during the rearing phase in hot climate pasture supplemented with protein sources

**DOI:** 10.1038/s41598-023-46420-5

**Published:** 2023-11-04

**Authors:** Lucimara Modesto Nonato, Luis Carlos Vinhas Ítavo, Camila Celeste Brandão Ferreira Ítavo, Vanessa Zirondi Longhini, Alexandre Menezes Dias, Gelson dos Santos Difante, Gabriella Jorgetti de Moraes, Rayane Chitolina Pupin, Paulo Henrique de Affonseca Jardim, Viviane Maria Oliveira dos Santos, Antonio Leandro Chaves Gurgel, Carolina Marques Costa Araujo

**Affiliations:** 1 Faculdade de Medicina Veterinária e Zootecnia, Universidade Federal de Mato Grosso do Sul, Campo Grande, MS 79070-900 Brazil; 2 Campus Professora Cinobelina Elvas, Universidade Federal do Piauí, Bom Jesus, PI 64900-000 Brazil

**Keywords:** Systems biology, Climate sciences

## Abstract

We hypothesized that the protein source in supplements and the insolation and ambient temperature changes in different seasons could cause changes in the skin of Nellore steers during the rearing phase on warm-climate pasture. The objective of this study was to evaluate the effects of replacing true protein (soybean meal) with NPN (extruded urea) in the supplement on the skin characteristics of steers grazing on Marandu grass pastures. Thirty-six Nellore steers with an average initial weight of 250.0 kg and 15 months of age were used. Skin biopsies were performed at three different times: summer, autumn, and winter. The protein source has effects on the length of the glandular portion, number of follicles, and gland area in steers during the rearing phase at different seasons of the year. In the summer, the skin presented a higher compact structure, while in the autumn and winter, the skin presented a sparser arrangement. Skin from steers that received soybean meal in supplement had a more significant number of follicles in the summer and a smaller area and length of sweat glands. The dermis thickness of steers supplemented with soybean meal was greater than that of urea supplemented. The epidermis thickness and dermis of the steers' skin were greater at the autumn (April). Sweat gland depth was greater in autumn (April) and winter (July) than in summer (December). The protein source in the supplement alters the skin characteristics by increasing of dermis thickness of Nellore steers during the growth phase. The insolation and ambient temperature changes in different seasons alter the skin structure by increasing the epidermis and dermis thickness, sweat glands depth, and glands area of the skin of steers during the growth phase.

## Introduction

The harmful effects of heat stress on farm animals have been studied since the early 2000s, and the debate and questioning about the impact of these effects on the physiological variables of animals continues today. The heat stress quantification and use of adequate methods to measure the physiological limits of exposure to stress in production animals has been the reason for several studies over time to optimize the responses of these animals so that they can express their genetic potential, and improve productivity^1^.

Zebu cattle are better able to regulate body temperature in response to heat stress than cattle of European origin^2^. The high ability to regulate body temperature during heat stress is the result of lower metabolic rates as well as a greater capacity for heat loss. Compared to European breeds, tissue resistance to heat flow from the body's core to the skin is lower for the zebu, while the sweat glands are larger. The properties of zebu skins increase heat loss by conduction and convection and reduce the absorption of solar radiation.

Sustainable ruminant production requires animals that are well adapted to heat, so there is a need for research efforts that can assist in the identification of thermotolerant breeds^3^. In the study by Tejaswi et al*.*^3^, animals of the Tharparkar breed, which are zebu natives of India, such as Ongole, preceptor of the Nellore breed, showed a greater ability to deal with heat stress during the summer. The authors indicated that the adaptation might be due to the non-enzymatic antioxidant melanin (melanin from black and brown pigments) that is abundantly present in the skin of Zebu cattle.

The generation of oxidative stress due to heat stress was greater in the lighter pigmented skin of crossbred cattle (*Bos indicus* × *Bos taurus*) than in darker pigmented skin, because oxidative stress in skin tissue increased with heat stress in cattle^4^. The heightened melanin expression in the skin of Zebu cattle (*Bos indicus*) likely results in more substantial pigmentation when compared to crossbred cattle. As a consequence, this increased pigmentation enhances their skin's protective capabilities, thereby mitigating oxidative stress in hot weather conditions. This factor is of paramount significance in explaining the superior heat tolerance exhibited by Zebu cattle in contrast to crossbred cattle.

Coat characteristics and sweating capacity are important for adaptability to heat stress and should be better studied and considered for selection for the genetic progress of adaptation in a tropical environment^5^. Souza et al*.*^6^ indicated that Zebu cattle developed adaptive characteristics to the natural environment and production systems in the tropics. Nascimento et al*.*^7^ studied skin samples from six *Bos indicus* breeds (Nellore, Cangaian, Gyr, Guzerat, Punganur, and Sindhi) during winter and summer, and the results showed seasonal variation in tropical conditions changes the morphological characteristics of the skin and sweat glands.

The characteristics of hair and skin, in addition to sweating, are important thermoregulatory mechanisms. Their association with endocrine, biochemical, and hematological reactions allows inferences about homeostasis. Those responses can be configured as a safety indicator of an animal's environmental adaptability^8^. According to Silveira et al.^9^, there are substantial relationships between the thermal environment and thermoregulatory responses, thyroid hormones, and coat characteristics of cattle, and concluded that zebu cattle are adapted to high temperatures; however, the thermal environment influences their thermoregulatory responses and coat morphology, consequently affecting their performance under grazing conditions.

The skin uses a quarter of the protein consumed daily by animals to produce new hair and epidermis, and those proteins are part of skin gland secretion and dermis repair^10^. Hence, when cattle are exposed to elevated temperatures and intense sunlight, the processes of skin and hair regeneration are likely to accelerate, potentially resulting in an elevated protein requirement for the upkeep of integumentary tissue. Consequently, supplying protein sources with enhanced digestibility and the capacity to bolster microbial protein production can serve as a dietary solution to enhance skin quality and bolster resilience against thermal stress induced by high temperatures.

Extruded urea is a product obtained by subjecting a mixture of starch (22% total digestible nutrients, TDN), urea (200% crude protein), and sulfur (3.2% S) to high temperature and pressure, resulting in starch gelatinization^11^. This processing method facilitates a gradual release of nitrogen in the rumen^11^. Moreover, it synchronizes nitrogen release with starch, promoting increased microbial protein production. Consequently, extruded urea can serve as a substitute for traditional protein sources, such as soybean meal, in ruminant diets^12,13^.

We hypothesized that the protein source in the supplement has effects on the ability of Nellore cattle adaptation to the sun at different seasons of the year. The objective was to evaluate the effects of replacing true protein (soybean meal) with non-protein nitrogen (extruded urea) in supplements on the skin characteristics of steers grazing on Marandu grass pastures during the rearing phase.

## Results

**Figure 1:**
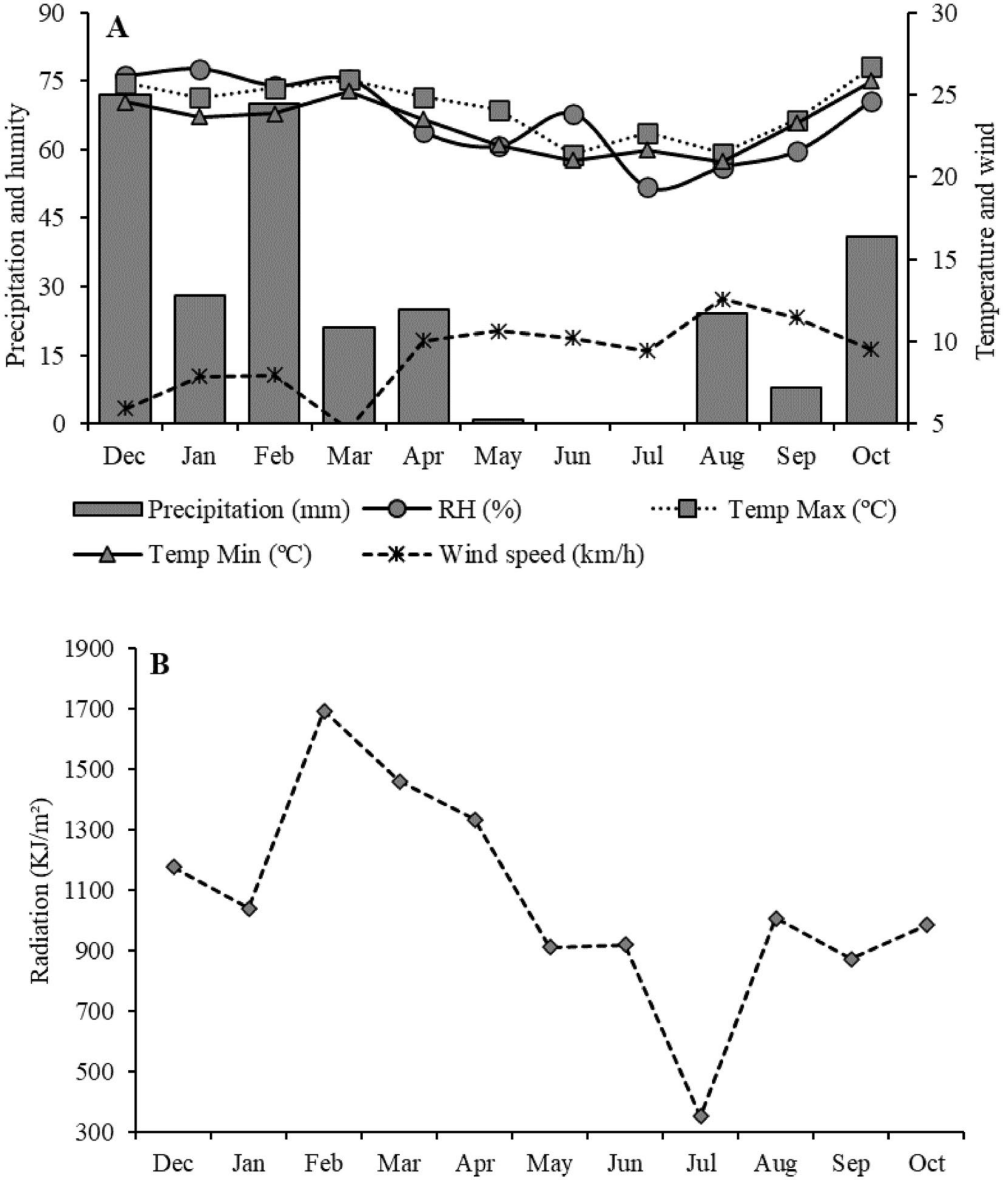
Meteorological (**A**) and global radiation (**B**) averages for Campo Grande, State of Mato Grosso do Sul, Brazil, calculated from the years 2017 and 2018, provided by the National Institute of Meteorology (INMET).

The weather condition altered the skin characteristics of Nellore steers during the rearing phase on warm-climate pasture, by prolonged exposure to high temperatures and insolation. The temperature presented a small annual amplitude, keeping the highest values in the summer period (25.5 ºC). Air humidity, rainfall, and global radiation also had the highest averages in summer (75.8%, 191 mm, and 1343.27 kJ/m^2^) (Fig. 1). The wind speed had the highest average in the winter season (10.92 km/h).

**Figure 2:**
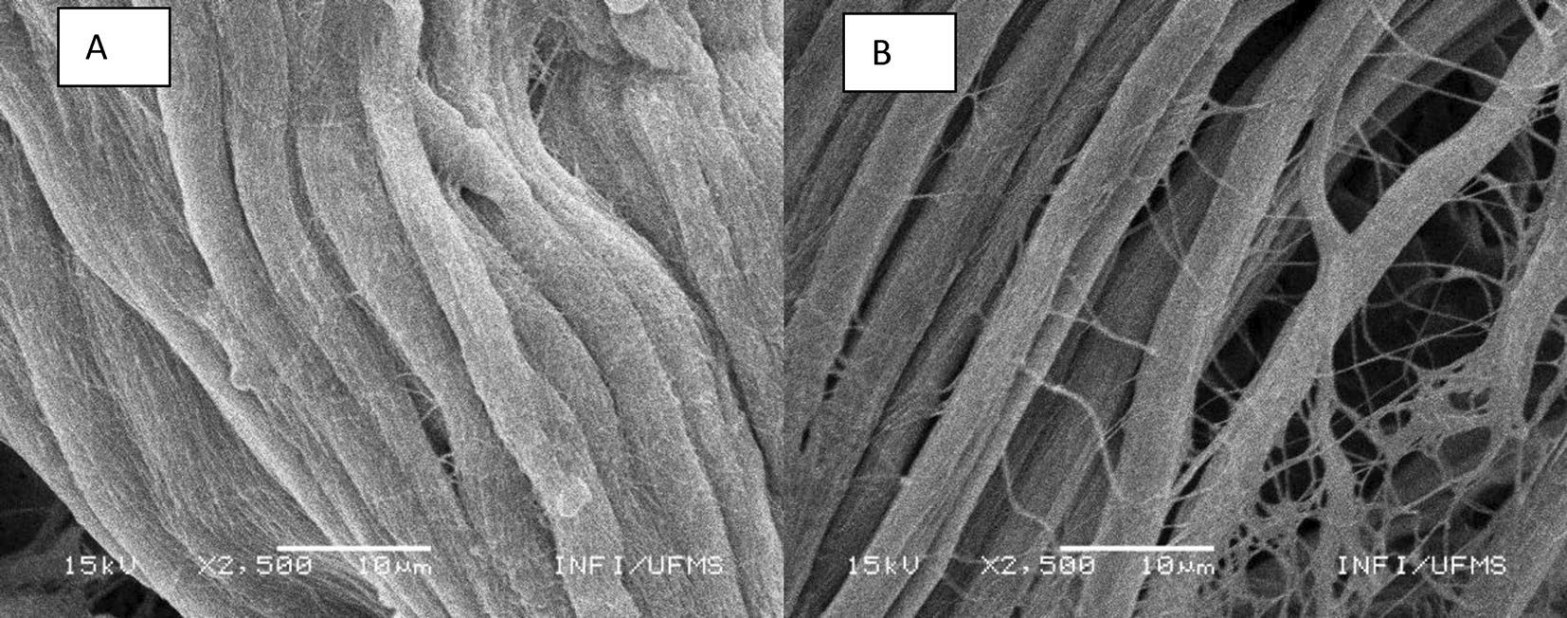
Morphology of steer skin submitted to Scanning Electron Microscope (SEM). (**A**) Steer skin in summer—the skin structure is more united. (**B**) Steer skin in winter—the skin structure is sparser.

The skin morphology of the steer evaluated by scanning electron microscope showed structural differences (Fig. 2). In the summer, the skin presented a higher compact structure, while in the autumn and winter, the skin presented a sparser arrangement.

**Figure 3:**
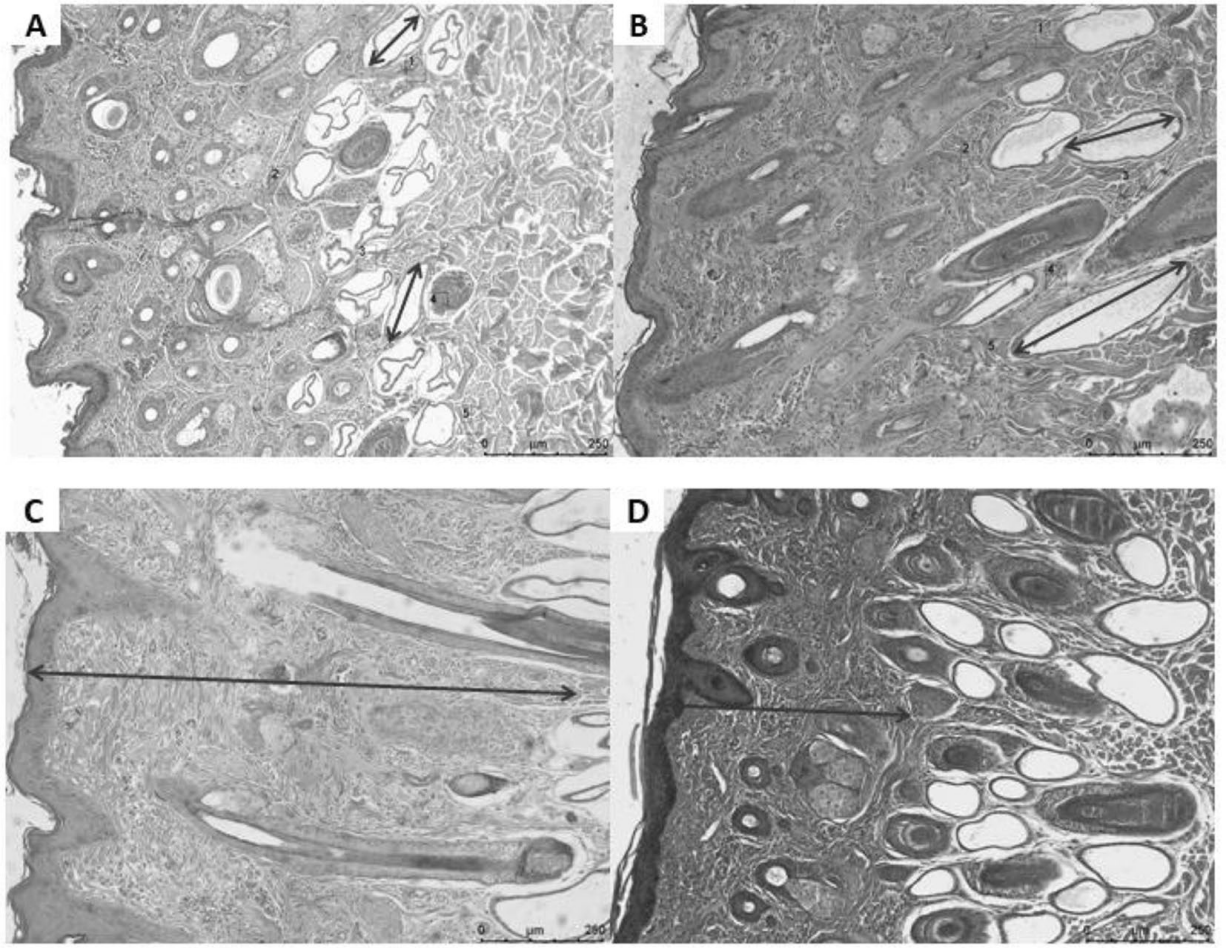
Number of follicles, area, length (**A** and **B**) of sweat glands, and thickness of the dermis (**C** and **D**) of steers as a function of the protein source in the supplement. (**A**) Steer skin that received soybean meal as a protein source in the supplement. Higher number of follicles (arrow) in summer and smaller area and length of sweat glands (arrow). (**B**) Steer skin that received extruded urea as a protein source in the supplement. Fewer follicles (arrow) in summer and greater area and length of sweat glands (arrow). (**C**) Steer skin that received soybean meal as a protein source in the supplement. Thickness of the dermis (arrow) greater than that of the animals that received extruded urea as a supplement. (**D**) Steer skin that received extruded urea as a protein source in the supplement. Thickness of the dermis (arrow) smaller than the animals that received soybean meal as a supplement.

The skin from steers that received soybean meal as a protein source in the supplement had a more significant number of follicles in the summer and a smaller area and length of sweat glands. The skin of steers that received extruded urea as a protein source in the supplement had fewer follicles in summer and greater area and length of sweat glands. The thickness of the dermis of steers supplemented with soybean meal was greater than that of steers supplemented with extruded urea (Fig. 3).

**Figure 4:**
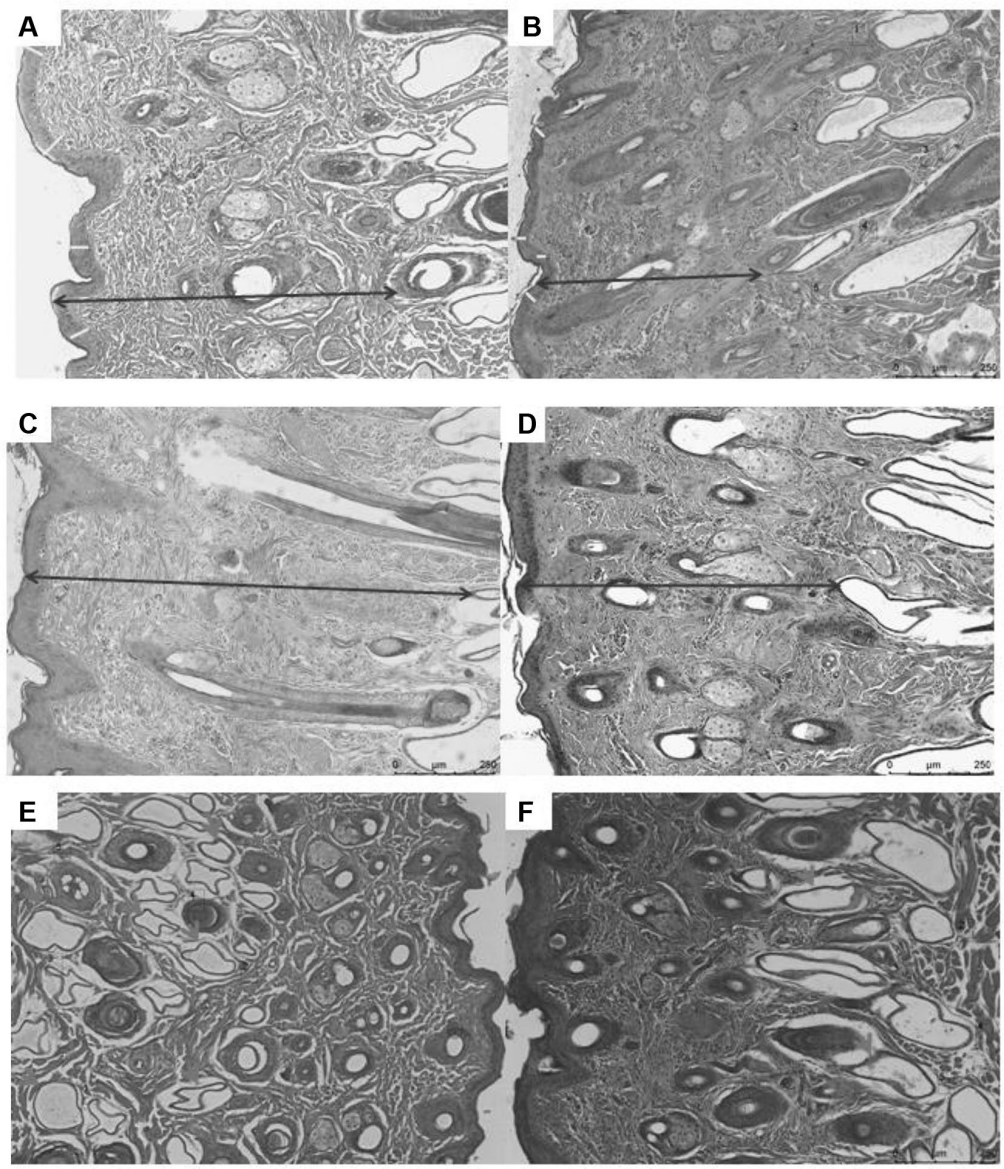
Thickness of the dermis (**A** and **B**), depth (**C** and **D**) and number (**E** and **F**) of sweat glands of steers as a function of the time of year. (**A**) Steer skin in winter. Thickness of the epidermis (arrow) and dermis (arrow) of bovine skin was higher in winter. (**B**) Steer skin in summer. Thickness of the epidermis (arrow) and dermis (arrow) of bovine skin was lower in summer. (**C**) Steer skin in winter. Sweat gland depth was greater in winter than in summer. (**D**) Steer skin in summer. Sweat gland depth was lower in summer than in winter. (**E**) Steer skin in summer. The number of sweat glands was higher in summer than in autumn and winter. (**F**) Steer skin in winter. The number of sweat glands was lower in winter and autumn than in summer.

There was an effect of the season of the year on the thickness of the epidermis and dermis. The thickness of the epidermis and dermis of the steers' skin was greater at the end of summer. Sweat gland depth was greater in autumn (April) and winter (July) than in summer (December). The number of sweat glands was higher in summer than in autumn and winter (Fig. 4).

There was a significant interaction (*P* < 0.05) between protein source × season of the year for the length of the glandular portion, number of follicles, and glands area. The supplement did not change the epidermis thickness, depth, number of glands, number of follicles, and area of sweat glands (Table 1).

**Table 1:** Skin characteristics of steers reared on pasture as a function of supplement and season.

	Protein source^a^		Season^b^			CV (%)	*P* value		
	Soybean meal	Extruded urea	Summer	Autumn	Winter		Protein source	Season	Interaction
Epidermis thickness (µm)	38.2^a^	37.2^a^	34.0^b^	41.8^a^	37.2^a^	27.46	0.2242	0.0001	0.0618
Dermis thickness (µm)	695.5^a^	666.6^b^	643.6^b^	716.8^a^	682.8^a^	17.98	0.0063	0.0001	0.2209
Length of the glandular portion (µm)	218.8^a^	223.1^a^	211.3^b^	215.9^b^	235.6^a^	43.78	0.6035	0.0416	0.0309
Sweat glands depth (µm)	709.8^a^	696.5^a^	688.6^b^	699.3^a^	688.8^b^	19.68	0.2632	0.0029	0.8843
Number of sweat glands	10.2^a^	10.6^a^	11.4^a^	9.5^b^	10.3^b^	28.45	0.1475	0.0001	0.0589
Number of follicles	19.6^a^	19.1^a^	20.8^a^	19.0^b^	18.2^b^	45.17	0.5222	0.0143	0.0001
Area of the glands (µm^2^)	5978.6^a^	5905.6^a^	4723.4^b^	5946.8^a^	7156.0^a^	63.08	0.8214	0.0001	0.0242

*CV* coefficient of variation.

^a^Means followed by different lowercase letters, differ from each other by the F test (*P* < 0.05).

^b^Means followed by distinct lowercase letters, differ from each other by Dunnett’s test (*P* < 0.05).

There was an effect of the season of the year for all skin variables. An increase was detected between the summer and winter for the average thickness of the epidermis, dermis thickness, and length of the glandular portion, depth, and glands area. On the contrary, there was a reduction between the summer and winter in the number of sweat glands and follicles.

There was a significant positive correlation between the thickness of the dermis and the thickness of the epidermis (r = 0.256); the thickness of the epidermis with the depth of the glands (r = 0.522), and between the thickness of the dermis and the depth of the glands (r = 0.463). Contrary, there was a significant negative correlation between room temperature and sweat gland area (r = − 0.330) (Table 2).

**Table 2:** Pearson’s correlation between the skin characteristics of steers reared on pasture.

	Dermis thickness	Sweat glands depth	Longer length sweat glands	Shorter length sweat glands	Number of sweat glands	Number of follicles	Gland area	Temperature
Epidermis thickness	0.256*	0.522*	0.064	0.012	− 0.300*	− 0.335*	− 0.001	− 0.118
Dermis thickness	1.0	0.463*	0.021	0.140	− 0.280*	− 0.131	0.041	− 0.127
Sweat glands depth		1.0	0.020	− 0.019	− 0.313*	− 0.267*	− 0.016	0.025
Longer length			1.0	0.273*	− 0.516*	− 0.528*	0.764*	− 0.132
Shorter length				1.0	− 0.172	− 0.109	0.657*	− 0.336*
Number of sweat glands					1.0	0.615*	− 0.450*	0.098
Number of follicles						1.0	− 0.391*	0.109
Gland area							1.0	− 0.330*

Correlations followed by * superscript indicate significance (*P* < 0.05).

## Discussion

The weather condition altered the skin characteristics of the steers through exposure to high temperatures and insolation. Wind speed can be beneficial because it aids in convective thermolysis. Air humidity and rainfall associated with high ambient temperatures can hamper heat dissipation and place animals under greater thermal stress during this period^14^. Likewise, Maia et al*.*^15^ observed that skin evaporation accounted for 80% of total evaporation in Holstein cows exposed to temperatures above 27 °C and was negatively correlated with air humidity. This confirms that the drier the environment, the greater the wear caused by the thermoregulation mechanism^15^. In addition, the association between the meteorological variables of the environment and the animals may have caused changes in skin morphology to maintain thermal balance.

The skin morphology of the steers had structural differences. This can be associated with the fact of the sweat glands are unevenly distributed throughout the body, with high density in some areas of the animal's body. In addition, there are differences in shape, depth, and size between the glands and the skin, which can affect sweating rates in different body parts^1,16,17^. In practice, it is not possible to determine the sweat rate for the entire surface, and it is necessary to determine a representative area for the average sweat rate. In this case, the average area of the central lateral side of the trunk, at the height of the ribs, presents the average value of the density of the sweat glands and the sweating rate in different areas of the body. Therefore, it was the place determined for the collection of the skins in the present study.

Weather conditions during the summer season resulted in a greater number of follicles and a smaller area and length of the sweat glands than at the end of summer and winter. Bhayani and Vyas^18^, observed an increase in the volume of sweat glands in winter compared to summer. The smaller volume of sweat glands indicates that they are at the peak of their functional activity, in contrast to the winter period when the volume increases, indicating a decrease in their functionality^1^. Likewise, Nascimento et al*.*^7^ observed changes in some parameters of the sweat glands in Nellore cattle that showed longer glandular section length in summer; however, the density, duct length, and sweat gland depth did not change among seasons. In cattle, the number of sweat glands is associated with the primary hair follicles, whose number remains stable in adult animals^19^. Therefore, the increase in the density of the follicles in the summer may be associated with the increase in the density of the sweat glands.

The impact of the nitrogen source on dermal thickness in our study highlights how the diet directly shapes the skin characteristics of the cattle. It is known that exposure to high ambient temperatures increases efforts to dissipate body heat, resulting in increased respiration rate, body temperature, and water consumption, decreasing feed consumption^20^. To date, no study has effectively evaluated whether the protein source influenced the skin characteristics of cattle. Some studies evaluated only the diet influencing the thermoregulation of animals. According to Guimarães et al*.*^21^, buffaloes submitted to an ambient temperature of 36ºC and 50% of concentrate in the diet fed, even with an increase in the sweating rate, was not enough to trigger major biological changes in the animals.

Regarding the seasons of the year, our study showed that the thickness of the epidermis and dermis of the skin of Nellore steers was greater at the end of summer. Carvalho et al*.*^22^ found a negative correlation between the layers of the epidermis and body temperature in cattle. The authors suggested that the number of layers of the epidermis might be involved in maintaining body temperature. In addition, as in the present study, we had the highest averages of maximum temperatures and radiation at the end of summer, this may explain the greater thickness of the skin at this time of year, as the animals were subjected to greater thermal stress since they were kept in tropical climate pastures. In addition, the thickness of the skin can interfere with the thermal exchange between the animals and the environment^11^.

The weather conditions at the end of summer resulted in the greatest depths of the glands. Our data differ from the other studies, which found the sweat glands located more superficially in summer and deeper in winter^1,7^. The greater depth of the glands at the end of summer can be explained by the fact that it is a period of transition between the seasons. However, in the winter, the functional activity of the sweat glands decreases and becomes deeper^23^. Studies suggest that the sweat glands in cattle are more superficial in the summer due to the sweating activity being more intense in this season of the year^24^. In addition, the number of sweat glands was higher in early summer than in autumn (April) and winter (July). This is due to the positive correlation between the length/diameter of sweat glands and their activity, which leads to changes in the number of sweat glands per unit of the skin surface^7^. It may explain the increase in the density of the glands at the beginning of summer in the present study because there was a positive correlation between the length measurements and sweat glands area. Furthermore, in the summer, there is an increase in the activity of the glands^1^.

Therefore, our hypothesis tested that the protein source in supplements and the insolation and ambient temperature changes in different seasons could cause changes in skin Nellore steers during the rearing phase on warm-climate pasture is confirmed partially by results. Although the different seasons change the skin characteristics, the protein source in the supplement does not promotes the same alterations, only in dermis thickness. Therefore, other nutrients in diet could be tested to evaluate their effects on adaption steers' skin managed in tropical environments, since we observed changes caused only by seasons.

## Conclusion

Using soybean meal as a protein source in the supplement modifies skin attributes by augmenting dermal thickness in Nellore steers during the growth phase. The insolation and ambient temperature changes in different seasons alter the skin structure by increasing the epidermis e dermis thickness, sweat glands depth, and glands area of the skin of Nellore steers during the growth phase.

## Material and methods

The study was carried out at the College of Veterinary Medicine and Animal Science (FAMEZ) of the Federal University of Mato Grosso do Sul (UFMS), Campo Grande, MS, Brazil (20° 26′ 50′′ S, 54° 50′ 21′′ W, altitude 417 m a.s.l.). The annual and regular rainfall is 1,625 mm (ranging from 1500 to 1750 mm), with a dry period of less than four months, corresponding to a water deficit of 350 to 500 mm.

All procedures involving animals in this study followed the guidelines of the National Council for the Control of Animal Experimentation and approved by the Ethics Committee in the Use of Animals of the Federal University of Mato Grosso do Sul (Protocol n. 805/2016). Study is reported in accordance with The ARRIVE (Animal Research: Reporting of in vivo Experiments) guideline^25^.

Thirty-six Nellore steers with an average initial weight of 250.0 kg and approximately 15 months of age were used. Animals were identified with plastic earrings, submitted to parasite control, and vaccinations according to the sanitary calendar. All animals were kept in Marandu grass pasture (*Brachiaria brizantha* Stapf cv. Marandu). The experimental area consisted of four paddocks (4.0 hectares each), with two paddocks per treatment with nine animals each, according to a completely randomized design.

Two supplements were formulated (Table 3), containing different protein source (extruded urea and soybean meal) with 41% and 26% of crude protein based on dry matter, respectively, for different seasons (Table 4). Supplements comprised mineral, grounded corn, and soybean meal or extruded urea. Nellore steers were supplemented with 0.45% of body weight (BW) during the rainy season (summer) and 0.7% of BW in the dry season (transition period summer–winter). The extruded urea used was Amireia-200® (Pajoara Ind. e Comércio Ltda. Campo Grande—Mato Grosso do Sul State, Brazil).

**Table 3:** Ingredients of supplements according to protein source and season.

	Treatment	
	Extruded urea	Soybean meal
Summer season supplement		
Grounded corn (g/kg DM)	78.00	19.53
Soybean meal (g/kg DM)	–	76.03
Extruded urea^a^ (g/kg DM)	17.55	–
Mineral mix^b^ (g/kg DM)	4.44	4.44
Summer–Winter transition season supplement		
Grounded corn (g/kg DM)	87.07	53.52
Soybean meal(g/kg DM)	–	43.62
Extruded urea^a^ (g/kg DM)	10.07	–
Mineral mix^b^ (g/kg DM)	2.86	2.86

^a^Extruded urea with 200% protein equivalent (Amireia-200^®^—Pajoara Ind. and Com. Ltda. Campo Grande-MS, Brazil).

^b^Warranty levels: Na: 100 g/kg; P: 88 g/kg; Ca: 188 g/kg; S: 22 g/kg; Mg: 8000 mg/kg; Zn: 3000 mg/kg; Cu: 1000 mg/kg; Co: 80 mg/kg; I: 60 mg/kg; Se: 4 20 mg/kg; F: 880 mg/kg.

**Table 4:** Composition of supplements according to protein source and season.

	Treatment	
	Extruded urea	Soybean meal
Summer season supplement		
Dry matter (DM, g/kg)	847.2	850.2
Ash (g/kg DM)	58.8	97.5
Organic matter (g/kg DM)	941.2	902.5
Crude protein (g/kg DM)	410.0	410.0
Neutral detergent fiber (g/kg DM)	139.5	272.8
Acid detergent fiber (g/kg DM)	34.2	105.7
Summer–Winter transition season supplement		
Dry matter (DM, g/kg)	915.7	901.4
Ash (g/kg DM)	39.5	56.7
Organic matter (g/kg DM)	960.5	942.4
Crude protein (g/kg DM)	267.5	267.5
Neutral detergent fiber (g/kg DM)	181.6	293.5
Acid detergent fiber (g/kg DM)	49.4	95.2

To evaluate the skin, a 1.0 cm^2^ trichotomy was performed in the left lateral region at the average height of the side of each steer. The fur coat thickness (CT) was measured from the perpendicular distance between the fur insertion points in the epidermis to the fur coat surface with a digital electronic caliper (model 799A-6/150, Starrett brand).

Skin biopsy^26^ was performed at three different times: summer (December), autumn (April), and winter (July). For the morphological evaluation of the skin, after asepsis with alcohol-iodine, a transverse fragment, with 1 cm in diameter, was collected from the left dorsal region (same site of the trichotomy), with the aid of a “Punch” type pourer, after administration of local anesthetic (2% lidocaine hydrochloride), subcutaneously. After removing the skin fragment, the perforation in the epidermis was sutured with a surgical stitch. Skin samples were fixed in 10% formaldehyde.

The samples were sent to the Histology Laboratory (FAMEZ/UFMS), dehydrated in ascending series of alcohol (70, 90, and 100%), treated with xylene and infiltrated in paraffin. The small blocks formed were cooled for 10 min to become more consistent and to facilitate microtomy. Three skin fragments from each animal were obtained by transverse incisions, and serial cuts (7 µm thick) were performed in each block. After that, they were deparaffinized and stained with hemotoxylin-eosin^27^.

The tissue sections were employed for quantifying the epithelial thickness (measured in linear µm) and dermal thickness (also measured in linear µm). For the sweat glands, we assessed their area (in µm^2^), depth (measured in linear µm), and the total count of sweat glands. Furthermore, we determined the number of hair follicles by utilizing the Image Analysis System (specifically, Image Pro-Express v.6.0) in conjunction with a micrometric scale, which was integrated with a light microscope from the Olympus brand (model BX40) using a 10 × objective.

The study of the morphology of the skin samples, as a function of the nutritional treatment, was carried out by scanning electron microscopy (SEM) in a JEOL equipment, model JSM 6380LV, at Physics Institute (INFI/UFMS). Samples in their original form (skin) were dispersed on double-sided carbon tape for fixation on top of a copper stub. The set was taken to the interior of the sputtering chamber (Denton Vacuum, Desk III model) for the deposition of a thin layer of gold on the surface of the dust particles to obtain a better resolution of the SEM image and an increase in conductivity. Sample power to avoid the effects of electronic charging that degrade image resolution. For the analysis, a voltage of 15 kV, working distance (WD) 12 mm and spot size 20 were used. In the context of observational analyses, measurements were collected, and images were captured at a magnification level of 2500 × to facilitate comparative morphological assessments.

The data analysis followed a completely randomized design implemented within a split-plot framework. In this setup, protein sources were designated as the primary plot, and the different time points across the year were assigned to the subplot. This analysis was carried out using PROC GLM within the SAS statistical package (SAS University Edition, Sas Institute Inc., Cary, CA, USA). To compare the means of different protein sources, we applied the F test, while for comparing means across different seasons, the Dunnett test was employed, with the commencement of summer serving as the control (initial sampling). Statistical significance was determined at a 5% level.

Additionally, Pearson’s correlation coefficients between variables were calculated using the PROC CORR procedure within SAS (SAS University Edition, Sas Institute Inc., Cary, CA, USA).

## Data Availability

All data generated or analyzed during this study are included in this published article.
